# Utilizing Crushed Limestone as a Sustainable Alternative in Shotcrete Applications

**DOI:** 10.3390/ma17071486

**Published:** 2024-03-25

**Authors:** Elamin Mutaz, Muawia Dafalla, Ahmed M. Al-Mahbashi, Mehdi Serati

**Affiliations:** 1 School of Civil Engineering, The University of Queensland, Brisbane, QLD 4072, Australia; m.mahmoud@uq.net.au (E.M.); m.serati@uq.edu.au (M.S.); 2 Department of Civil Engineering, King Saud University, Riyadh 11362, Saudi Arabia; aalmahbashi@ksu.edu.sa

**Keywords:** shotcrete, tunnel support, crushed limestone, crack initiation

## Abstract

Solving the challenges facing the mining industry is crucial for shaping the global attitude towards clean energy technologies associated with critical minerals extracted from depth. One of these challenges is the well-known explosion-like fractures (rockbursts) or spalling failures associated with the initiation of internal cracks. To prevent such catastrophic failure, shotcrete, as a cement grout, is widely used in tunnel support applications. In areas where the tunnels are constructed within the limestone strata using tunnel boring machines (TBM), drilling, and/or blasting, millions of cubic meters of crushed limestone (CL) in powder form are extracted and landfilled as waste. Given the fact that natural sand consumption as a raw material in the construction industry exceeds previous records, recycling of such excavation material is now becoming increasingly needed. From this perspective, this study aims to utilize crushed limestone as a potentially sustainable alternative to natural sand in shotcrete applications in deep tunnels. Accordingly, several strength characterization and crack initiation determinations through various stress–strain-based models were carried out on cylindrical samples containing different proportions of crushed limestone. By increasing the crushed limestone content in the shotcrete mix, the crack initiation stress (as a measure of the in situ spalling strength) increased as well. The results suggest that the crushed limestone has good potential to replace the natural sand in the shotcrete mixture used in tunnel support applications.

## 1. Introduction

### 1.1. Background and Overview

**Figure 1:**
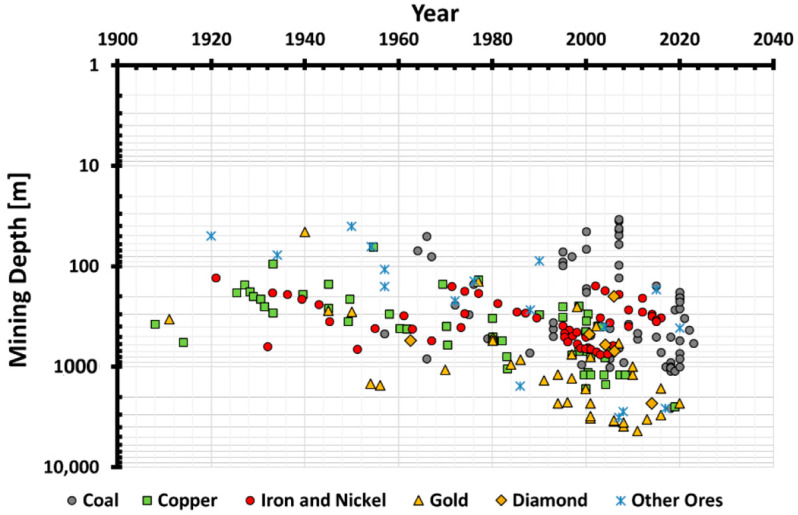
Mining depth variation over the past century for different ores [7].

Since the early ages, resources and minerals have been exploited by humans in an attempt to survive and thrive. Following the industrial revolution in the 19th century, the exploitation pattern of mineral resources gradually increased, and the minerals at superficial depths were exhausted, which triggered the trend towards deeper exploration. In today’s world, mining depths in excess of 1000 m have become usual in coal and metal mines around the globe [1,2,3]. In the case of non-ferrous metal mines, mining operations have delved even deeper, to 4000 m and beyond, e.g., the Mponeng gold mine and the TauTona or Western deep-level gold mine in South Africa [4]. Booms in the mining industry are due to access to state-of-the-art drilling techniques and instrumentation methods. Figure 1 is a graphical representation of mining depth variation over the past century for different ores and commodities [4,5,6].

Rock is a heterogeneous material that can have a large number of discontinuities with non-elastic behavior. At shallow depths where in situ stresses are not large, erosion, anisotropy, the presence of discontinuities, and other similar local effects can introduce a large variation in rock elasticity, density, and thermal expansion properties. But, as mines go deeper, the underground rock becomes subjected to a more complex environment of high-stress conditions and high temperatures where rock porosity considerably decreases. Consequently, rock behavior at depth could be very different from that near the surface. The tensile fracturing of hard and competent rocks in deep tunnels under high geo-stresses can be abrupt and violent, leading to shattering without any warning. Such explosion-like fractures in the field are commonly known as stress spalling, slabbing, or rockbursting, caused by the buckling of rock layers detached by the tensile fractures. Several extremely intensive rockburst incidents have been recorded in locations around the globe: the Jinping II Hydropower Station in China, Ontario’s Kirkland Lake mines in Canada, the Coeur d’Alene district in USA, the Sourva-Vietas hydro-power tunnel in northern Sweden, the Miike Coal Mine in Japan, and also in other countries [8,9,10,11,12,13]. The earliest definition of spalling was provided by Terzaghi as popping rock [14]. Since then, many other definitions of spalling failure have been introduced. According to Martin and Christiansson [15], spalling failure is the formation of stress-induced slabs on the boundary of underground excavation. Conversely, according to Gong et al. [16], spalling is closely related to the propagation and coalescence of internal tension cracks surrounding the rock. Nevertheless, spalling can trigger rockburst by forming thin rock slabs which facilitate the sudden release of energy and, therefore, cause violent rock ejection [17]. Like rockburst, spalling failure has been reported globally in numerous mining sites in Canada, North America, India, Sweden, Finland, Switzerland, Iran, China, Australia, and other countries [18,19,20,21,22,23,24,25].

**Figure 2:**
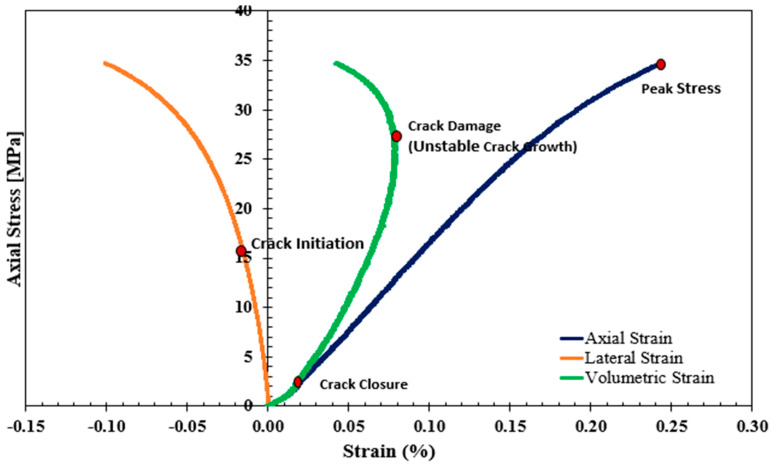
Crack closure, crack initiation, and crack damage identification based on the stress–strain response [7].

**Figure 3:**
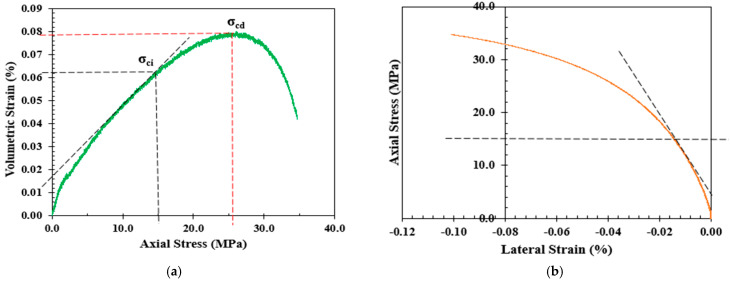

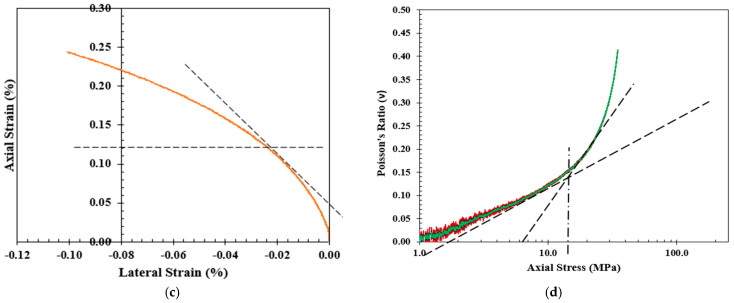
Determination of CI under uniaxial loading condition via (**a**) volumetric strain method, (**b**) lateral strain method, (**c**) extensional strain method, and (**d**) Poisson’s ratio method [7].

Shallow spalling, where the depth of spalling is less than 25 cm, is generally associated with tensile failures, leading to the generation of large flakes with flat cutting faces and sharp edges. In addition, minimal fines are usually observed and conchoidal fracture surfaces are formed that follow the stress path around the excavation. In deep spalling, where the depth of spalling could be more than 1 m, a more complex combination of tensile and shear failures is commonplace, forming V-shaped (large dog-eared) notches in the immediate excavation boundary. However, regardless of the spalling strength, the failure is believed to occur at stress levels corresponding to the initiation of internal cracks in rock. From this perspective, a crack initiation stress (CI or $\sigma_{ci}$) concept, defined as the onset of microcracks and the nucleation of grain-scale load-induced stress following the closure of pre-existing cracks, was introduced as a predictor of spalling failure [13]. Based on the stress–strain response of underground rock under compression loading, four distinct stages can be readily identified: closure of pre-existing cracks, crack initiation, unstable crack growth, and peak strength stages, as presented in Figure 2. Below and a bit above the CI stress threshold, the deformation is still under elastic behavior, where material elasticity (i.e., elastic modulus E and Poisson’s ratio υ) can be determined. Hence, many available crack initiation models were developed based on the stress–strain response of underground rock as summarized in Table 1 and detailed in Figure 3. Moving up towards the unstable crack growth, plastic deformation takes place. Following the closure of pre-existing cracks, stress-induced fractures/cracks start to initiate at approximately 30% to 60% of the unconfined compressive strength regardless of the loading or environmental conditions. Hence, the concept of a crack initiation ratio (CIR), defined as the ratio of the CI stress threshold to the peak strength, was proposed as a dimensionless predictor to better characterize spalling failure [15]. Even though $\sigma_{ci}$ and CIR might seem similar as predictors for spalling failure, they can show contradictory trends. Based on intensive data collected from the literature, Mutaz et al. [13] summarized the effect of material elasticity, porosity, and tensile strength on both $\sigma_{ci}$ and CIR—see also Table 2.

### 1.2. Shotcrete and Grout in Tunneling

The use of grout and shotcrete is a common practice in the tunnel industry. Grouts normally consist of sand and cement, while shotcrete is a concrete material designed to be sprayed onto a surface, as opposed to being poured. Rock support systems play a pivotal role in harnessing the intrinsic strength of rock masses, ensuring self-supporting tunnel walls [26]. These systems employ a combination of techniques, such as dowels, tensioned rock bolts and cables, shotcrete, mesh, and steel sets, to bear the loads from individual rock blocks separated by structural discontinuities or loosened rock zones [26]. The installation of rock support systems is integral to enhancing the self-supporting capacity of the rock mass. Predicting the precise load acting on these support systems is challenging, necessitating design methodologies based on classification systems (e.g., Q-method and Rock Mass Rating System—RMRS), numerical analysis, and tunnel geometry [27].

**Table 1:** Stress–strain-based models for crack initiation determination.

Model	Crack Initiation Stress ($\sigma_{ci}$) Determination
Volumetric Strain Method [28]	The volumetric strain ($\varepsilon_{v}$) is the summation of axial ($\varepsilon_{1}$) and lateral strains ($\varepsilon_{2}$) and ($\varepsilon_{3}$) in the direction of the major, intermediate, and minor principal stresses, respectively. The crack initiation ($\sigma_{ci}$) in this model is defined as the point where the volumetric strain curve deviates from its linearity.
Lateral Strain Method [29]	This model suggests that the lateral strain is more sensitive than axial strain to the growth of cracks before the onset of unstable crack growth on the stress–strain response. Therefore, the crack initiation ($\sigma_{ci}$) is determined as the point where the lateral strain deviates from the linearity of the axial stress ($\sigma_{1}$) vs. lateral strain curve.
Extensional Strain Method [30]	The extensional strain method was proposed suggesting that the extensional strain could be determined experimentally by plotting the lateral strain versus axial strain. Therefore, the crack initiation ($\sigma_{ci}$) is determined as the point where the axial strain tends to deviate from its linearity on the axial strain versus lateral strain curve.
Poisson’s Ratio Method [17]	In this model, Poisson’s ratio is plotted against the log of axial stress, and the intersection of the tangents to the linear portions of the curve are determined to calculate the crack initiation stress ($\sigma_{ci}$).

**Table 2:** Variation in $\sigma_{ci}$ and CIR with elasticity, porosity, and tensile strength [7].

Parameter	Crack Initiation Stress ($\sigma_{ci}$)	Crack Initiation Ratio (CIR)
Elastic Modulus (E)	Increases with an increase in elastic modulus	Independent of elastic modulus
Poisson’s Ratio (υ)	Independent of Poisson’s ratio; at the same Poisson’s ratio, $\sigma_{ci}$ is a function of rock type	Independent of Poisson’s ratio
Porosity (η)	Decreases with an increase in porosity	Decreases with an increase in porosity
Tensile Strength $\left(\sigma_{t}\right)$	Increases with an increase in tensile strength	Increases with an increase in tensile strength

**Figure 4:**
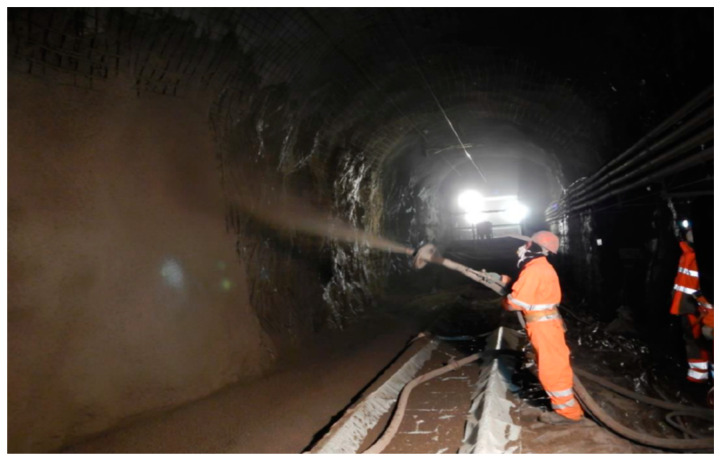
Shotcrete spraying around a tunnel sidewall.

Shotcrete, also known as sprayed concrete, stands as a widely utilized material in the construction industry, serving as a prominent lining technique in diverse mining and civil applications [31,32]. Its versatility allows it to conform to various civil structure shapes and adhere to uneven surfaces, offering effective and cost-efficient ground support with rapid application [31,33]. Shotcrete consolidates loose rock masses surrounding excavations, penetrating small perimeter fractures to confine rock mass between adjacent reinforcement elements [34,35]. The thickness of the shotcrete layers typically ranges between 2 and 30 cm, depending on the purposes and applications. Shotcrete can be applied in different ways: (i) dry method as a dry-sprayed concrete and (ii) wet method as a wet-sprayed concrete, as shown in Figure 4. The New Austrian Tunneling Method (NATM) provides some innovative techniques like the sprayed concrete lining (SCL) method, which adapts to the shape of the tunnel profile, accommodating irregularities and variations in the rock mass [36]. Nowadays, shotcrete application is mainly performed through remotely controlled spraying machinery, which increases the safety concerns of laborers working under potentially unsafe, unsupported rock [31]. However, it is crucial to recognize that shotcrete linings may deform when exposed to dynamic loadings, such as energy released during rockbursts or spalling [37]. Consequently, potential shotcrete failure mechanisms have been identified in the literature, including adhesion loss, flexural, direct shear, punching shear, compressive, and tensile failure, with their prevalence dependent on rock mass behavior at failure [35,38].

In deep geological structures parallel to tunnel walls or forming wedges, violent failures may extend beyond 1.0 m in depth, necessitating reinforcement measures to dissipate energy. The extent of reinforcement required varies based on factors like pre-existing cracks and the size of ejected blocks [39]. Larger mobilized block sizes demand more reinforcement, whereas smaller block sizes can often be effectively managed through a combination of shotcrete and mesh. Nonetheless, shotcrete functions similarly to mesh in preventing the disintegration of rock fragments within the excavation [26]. Ultimately, the performance of the support system in stabilizing the excavation is paramount, ensuring that the load transferred to reinforcement elements can be sustained effectively [40].

**Figure 5:**
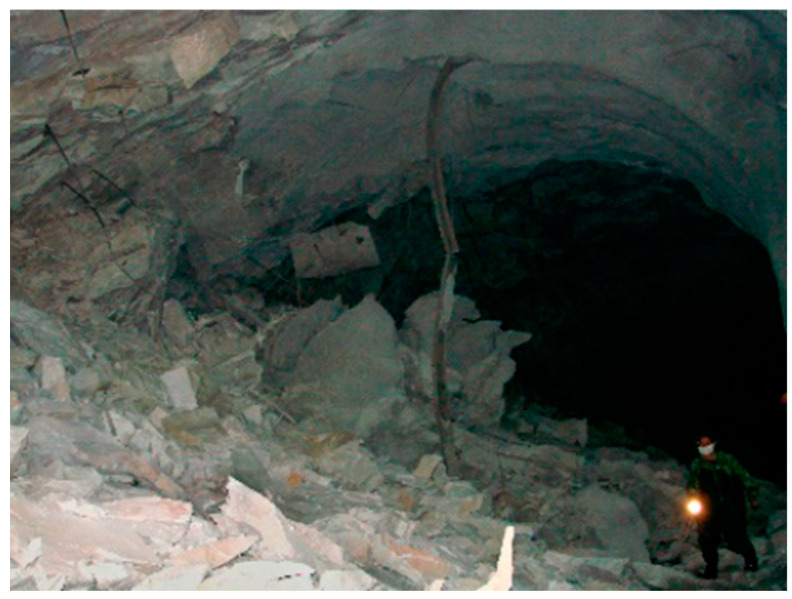
Supporting systems failure in underground tunnels following violent rockbursting and spalling due to instability of the reinforcement elements [46].

Shotcrete has been employed in tunneling to prevent rock fallout caused by spalling failures since the 1960s in various global projects, such as the Lieraasen railway tunnel west of Oslo and the Jinping II hydropower station tunnels [41,42]. In Australia, where weak rock mass quality often poses challenges, shotcrete has gained significant attention in the tunnel and mining sectors, with substantial investments exceeding AUD 42 billion [43]. Despite the successful application of shotcrete in tunneling in areas where poor rock mass quality is encountered—specifically, in deep mines that are prone to violent rock failure in the form of rockburst and/or spalling—rock fragments breaking through the shotcrete layer were observed in some locations, such as the bending deformation and ejection of rock fragments and shotcrete in the Jinping II hydropower station and the distortion and spalling of shotcrete in the Liujiazhuang tunnel constructed in coal rich weak rock stratum during 2012 [35,42,44,45]. Figure 5 presents supporting systems, including shotcrete, that fail after rockbursting or spalling. Despite the successful applications and simulations of shotcrete in supporting tunnels that are prone to spalling or bursting, limited research exists on shotcrete behavior at the crack initiation threshold—an essential predictive measure for in situ spalling failure.

Shotcrete is formed by mixing cement with sand and water and is used in common applications incorporating admixtures. It finds predominant use in soil improvements—specifically, jet grouting, tunnel lining, crack repair, and various concrete applications [47,48]. As the demand for tunneling operations continues to rise, shotcrete has become increasingly vital for ground support during tunneling and mining activities. For instance, over 800,000 m^3^ of shotcrete were employed in Australia in 2018 alone, used in diverse civil projects, including basements, swimming pools, and embankments [43]. A key concern in the construction of sustainable infrastructure is the continuous diminution of readily available construction aggregates including sand [49]. Due to the rapid consumption of natural sand as a raw material in construction and other activities, it has become the second-most widely consumed natural resource on planet Earth, after freshwater [50]. The estimated annual consumption of sand and gravel, largely used in construction activities, is between 32 and 50 billion tons [50,51]. From an environmental perspective, the process of extracting and transporting aggregates (including sand) generates a carbon footprint, posing a threat to the ecosystem, as noted by [52]. Therefore, on a global scale, the attitude towards sustainability has led to an increasing demand for alternative, environmentally friendly construction materials [53].

The estimated annual accumulation of industrial waste stockpiled, landfilled, and disposed of in vast areas worldwide exceeds a staggering amount, reaching thousands of tons [54]. The recycling of these waste materials has garnered significant global attention and engagement at the highest governmental levels. The literature is replete with many wastes recycled and utilized as construction materials in terms of replacements to sand in concrete mixtures such as crushed waste glass, ground granulated blast furnace slag (GGBS) as a byproduct of iron and steel production, marble waste, and organic waste materials like rice husk ash, wood ash, corncob granules, and wheat straw [50,55,56,57].

Notably, the utilization of wastes as construction materials extends to materials like crushed powder generated during tunnel excavation for subways and transportation networks. In Saudi Arabia, for instance, the estimated volume of crushed limestone produced during tunnel boring machine (TBM) activities exceeds 3 million cubic meters [48]. The physical and chemical characteristics of crushed limestone vary depending on the parent rock, typically featuring particle sizes of 75–80 µm, calcium oxide quantities of 40–60%, and significant silicon oxide content, making it a potential substitute for natural sand in various applications [58,59]. Utilization of such waste materials spans various geotechnical applications, including fluid control in clay liners, structural concrete aggregate, an alternative to commercial bentonite, and cement grout [48,58,60,61,62,63]. Some studies have emphasized the role of crushed limestone in enhancing the compressive strength, durability, and workability of concrete [60]. However, there remain areas of concern that require further research: assessing long-term durability, comprehensively evaluating environmental impact, exploring limestone variability, and investigating the structural behavior of concrete with crushed limestone replacements.

Nonetheless, despite successful applications of limestone powder as an alternative to cement grout, challenges persist in using crushed limestone as a grouting material in highly stressed tunnels that are prone to spalling or bursting. Furthermore, limited research is available regarding the impact of crushed limestone on crack initiation thresholds, a crucial aspect of assessment. This focal point in our study highlights the importance of addressing these gaps and contributes to advancing sustainable construction practices.

## 2. Experimental Setup and Methodology

### 2.1. Material Characteristics and Sample Preparation

**Figure 6:**
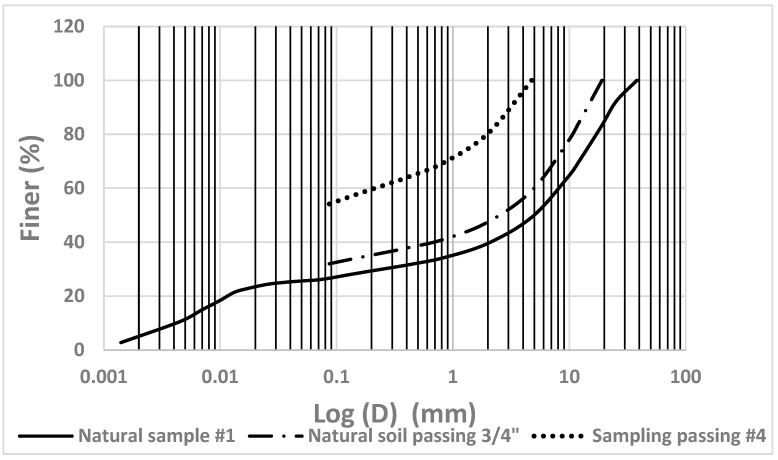
The grain size distribution of crushed limestone material.

**Figure 7:**
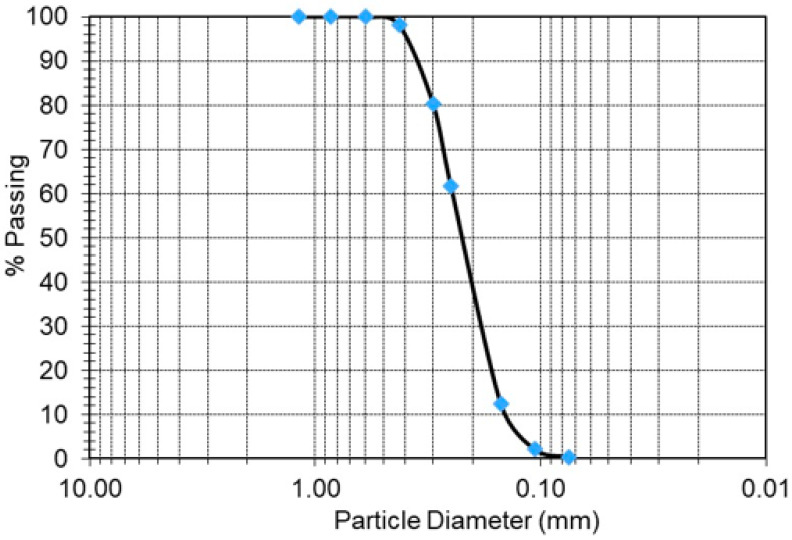
The grain size distribution of sand.

Shotcrete as a cement grout is mainly formed by mixing cement and sand in the presence of water and in some applications, admixtures are added. Given the boom in the tunneling industry in Riyadh, Saudi Arabia, millions of cubic meters of crushed limestone (CL) powder were produced by tunnel boring machines (TBMs) and stockpiled as waste. Therefore, the CL powders used in this study as a replacement for sand in shotcrete applications were brought from tunneling sites and tested at the Bugshan Research Chair in Expansive Soils (BRCES) within the School of Civil Engineering at King Saud University in Riyadh. Three cement-to-CL ratios of 1:1, 1:1.2, and 1:1.4 by dry weight were considered in this study. A control mixture of 1:1 cement to sand ratio by dry weight was prepared to assess the efficiency of sand replacement. Table 3 presents the designated shotcrete mixtures used in this investigation. A water–cement ratio (*w*/*c*) of 0.6 per dry weight was adopted for the cement with CL. However, the *w*/*c* ratio of the control mixture was maintained at 0.5 by dry weight to achieve the required uniformity and workability. The cement used in this study was a local Portland cement produced by Yamama Cement Co in Alkharj plant branch. A locally produced, poorly graded sand, with coefficients of uniformity and concavity of 1.713 and 0.945, respectively, was used in this study according to ASTM D2487–017 [64]. It is worth mentioning here that crushed limestone can also be described as sand if the grain size limitation is met. However, in this study, we used the term crushed limestone to refer to a material obtained by crushing limestone. This can fit the sand specification and can also include fines or coarse material beyond the sand limits. Figure 6 and Figure 7 present the grain size distributions for the crushed limestone and sand used in this study, respectively.

**Figure 8:**
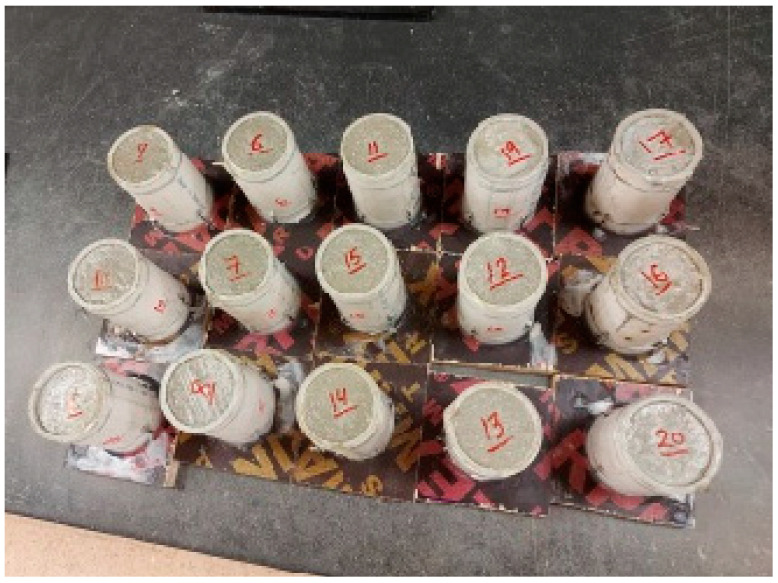
UCS shotcrete specimens prepared on 100 mm height and 50 mm diameter molds.

The CL powders that passed Sieve 4.75 mm size were selected for this study. According to the ISRM’s suggested methods for determining the uniaxial compressive strength and deformability of rock materials (1979), the diameter of the UCS specimen should be at least 10 times the largest grain size in the rock, and the test specimen should have a height-to-diameter ratio of 2.5–3.0. The cement grout samples were prepared on fabricated cylindrical molds of 100 mm height and 50 mm diameter (slightly deviating from the ISRM suggestion), as shown in Figure 8. To facilitate the extrusion of the samples, the inner walls of the molds were lubricated using an oil substance. The samples were layered into five layers and each layer was compacted using a steel rod. To maintain a smooth finished surface, an edge knife was used.

### 2.2. Crack Initiation Setup

**Figure 9:**
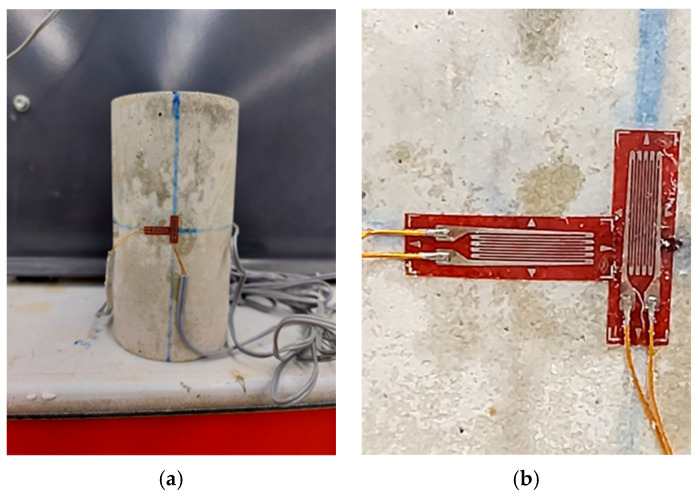
Strain and elastic parameter measurements: (**a**) strain gauge arrangements around the UCS sample, (**b**) close-up view of two-element strain gauges.

**Figure 10:**
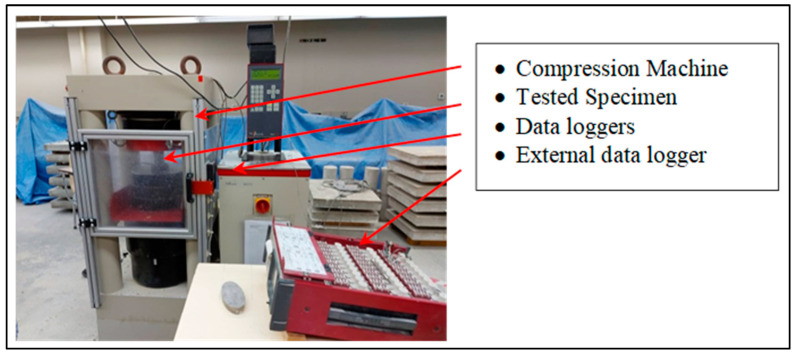
A Toni/Technic compression loading frame used in the study [48].

**Figure 11:**
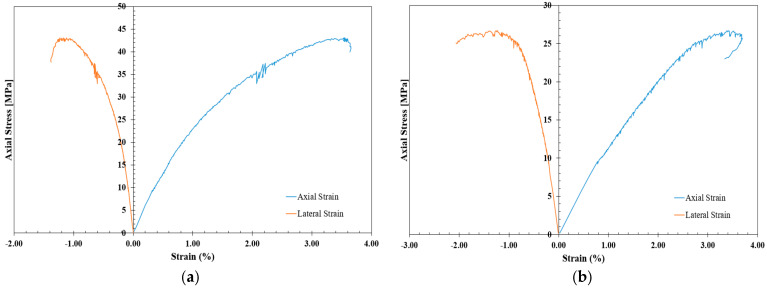

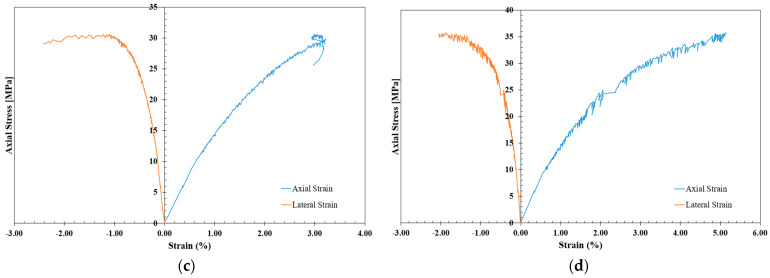
Stress–strain curves of the shotcrete samples with different mixtures: (**a**) 1 Cement:1 Sand, (**b**) 1 Cement:1 CL, (**c**) 1 Cement:1.2 CL, and (**d**) 1 Cement:1.4 CL.

The study of stress–strain behavior in cement grout is of great importance in designing safe shotcrete to protect against rock spalling. To determine the vertical and lateral strains required for crack initiation determination through stress–strain-based methods, strain gauges containing vertical and horizontal elements were arranged so that the UCS sample had four in total, equally spaced in the middle of the cylinder, as shown in Figure 9. The UCS tests were carried out using a Toni/Technik compression loading frame with a maximum capacity of 3000 kN and external data loggers, as shown in Figure 10. The loads were applied gradually at a rate of 1 mm/min until the failure was attained. Then, stress–strain curves of all designated shotcrete samples were plotted, as shown in Figure 11.

One of the earliest methods to determine the CI is the volumetric method proposed by Brace et al. [28]. Brace and co-authors observed that the onset of dilatancy can be readily determined when the volumetric strain curve deviates from its linearity [65]. Therefore, a volumetric strain ($\varepsilon_{v}$) as the summation of axial ($\varepsilon_{1}$) and lateral strains ($\varepsilon_{2}$) and ($\varepsilon_{3}$) in the direction of the major, intermediate, and minor principal stresses, respectively, was introduced. Under the uniaxial loading conditions, both $\varepsilon_{2}$ and $\varepsilon_{3}$ are equal; hence, the volumetric strain can be determined as follows:

(1) $$\varepsilon_{v}=\varepsilon_{1}+{2\varepsilon }_{2}$$

Lateral strain exhibits a higher sensitivity to crack growth compared to axial strain before reaching unstable crack growth in the stress–strain response [66]. Therefore, Lajtai [29] proposed the lateral strain method, which suggests that axial strain maintains a linear pattern from crack closure to the onset of unstable crack growth. In other words, the crack initiation stress is the vertical applied stress when the lateral strain deviates from its linearity on the axial stress versus lateral strain curve, as presented in Figure 3b.

The extensional strain method was proposed by Stacey [30] to determine the crack initiation based on stress-induced failures that occurred in gold mines in South Africa. In this method, the extensional strain is determined by plotting both the axial and lateral strains. The point where the axial strain tends to deviate from its linearity is the corresponding point of crack initiation on the stress–strain response curve, as shown in Figure 3c.

Diederichs [17] proposed a CI model in which Poisson’s ratio (υ) is plotted against the log of the axial stress ($\sigma_{v}$) during a UCS test, where the intersection of the two tangents to the linear portions of the curve are determined to calculate the CI level, as shown in Figure 3d. The Poisson’s ratio is simply the ratio between the horizontal $\left(\varepsilon_{h}\right)$ and vertical $\left(\varepsilon_{v}\right)$ strains measured by both elements of the strain gauges. However, determining the strains and Poisson’s ratio using four equally spaced two-element strain gauges, rather than using axial and circumferential extensometers that cover the entire perimeter of the samples, can overestimate the results by up to 20% [7].

## 3. Crack Initiation Determination

**Figure 12:**
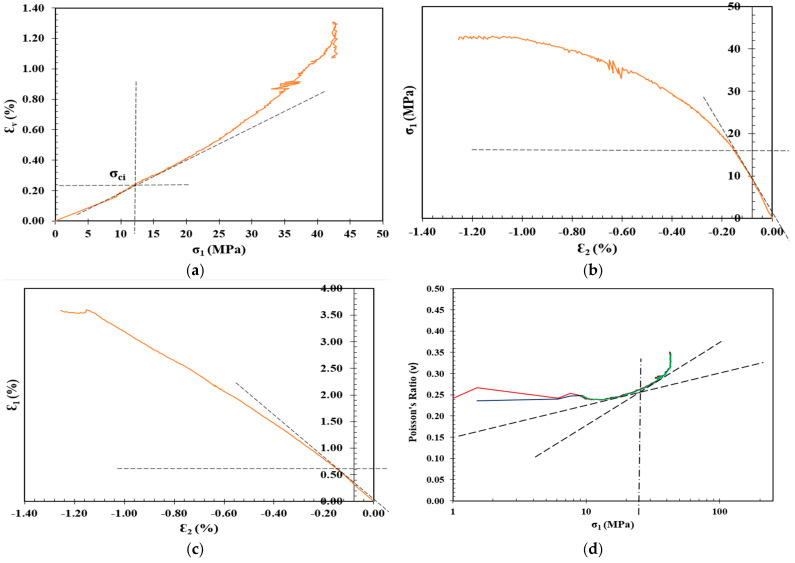
Determination of CI for control shotcrete mixture through the (**a**) volumetric strain method, (**b**) lateral strain method, (**c**) extensional strain method, and (**d**) Poisson’s ratio method.

**Figure 13:**
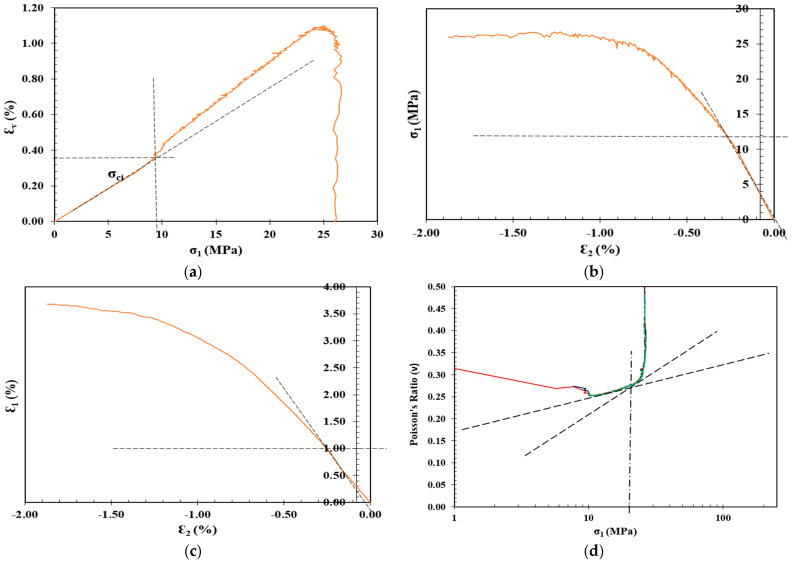
Determination of CI for 1 Cement: 1CL shotcrete mixture through the (**a**) volumetric strain method, (**b**) lateral strain method, (**c**) extensional strain method, and (**d**) Poisson’s ratio method.

**Figure 14:**
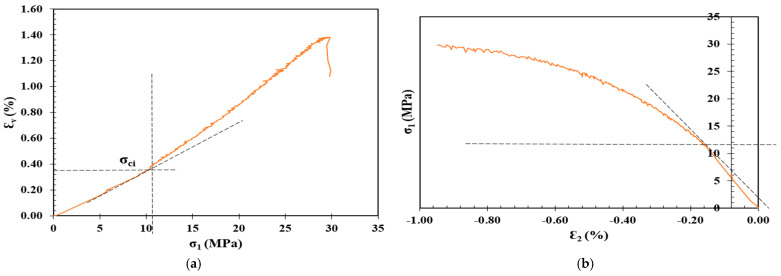

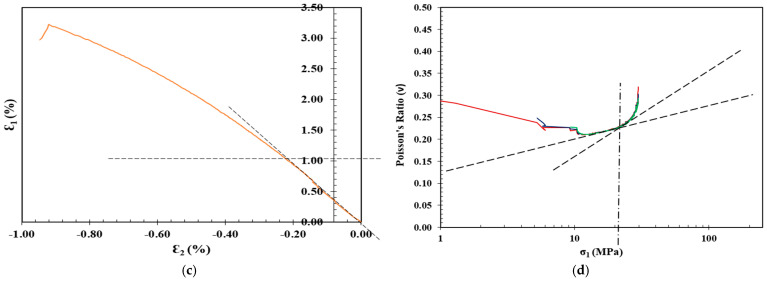
Determination of CI for 1 Cement: 1.2 CL shotcrete mixture through the (**a**) volumetric strain method, (**b**) lateral strain method, (**c**) extensional strain method, and (**d**) Poisson’s ratio method.

**Figure 15:**
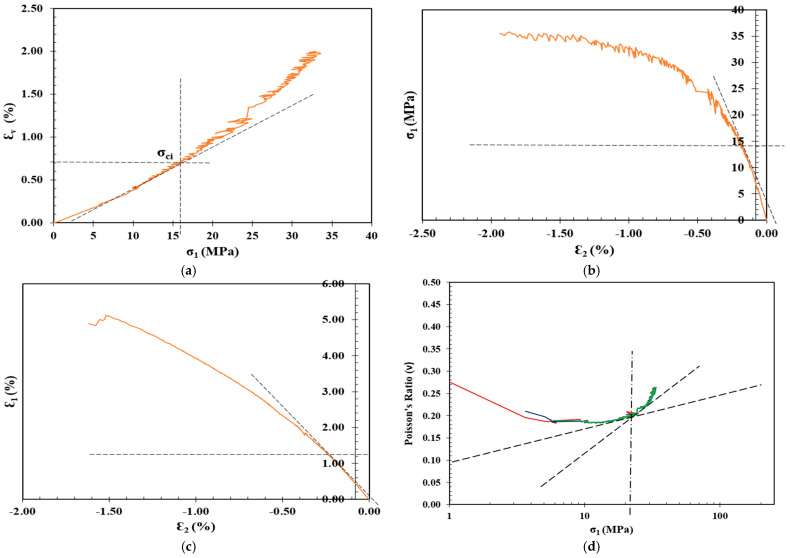
Determination of CI for 1 Cement: 1.4 CL shotcrete mixture through the (**a**) volumetric strain method, (**b**) lateral strain method, (**c**) extensional strain method, and (**d**) Poisson’s ratio method.

The determination of $\sigma_{ci}$ for the shotcrete samples in Table 3 was carried out through the following stress–strain methods: volumetric strain method, lateral strain method, extensional strain method, and Poisson’s ratio method, as summarized in Table 4, along with the CIR, standard deviation (SD), and coefficient of variation (CoV), and plotted in Figure 12, Figure 13, Figure 14 and Figure 15.

## 4. Effect of Crushed Limestone on the Strength and Crack Initiation

In the realm of construction materials and structural integrity, the application of cement-based grouts plays a pivotal role in ensuring the durability and safety of various infrastructural elements, such as tunnels and underground structures. The assessment of their performance, particularly in the context of strength and crack initiation, is of paramount importance. This study investigates the utilization of crushed limestone as an alternative to sand in shotcrete formulations, exploring its influence on the critical parameter of the crack initiation threshold.

**Figure 16:**
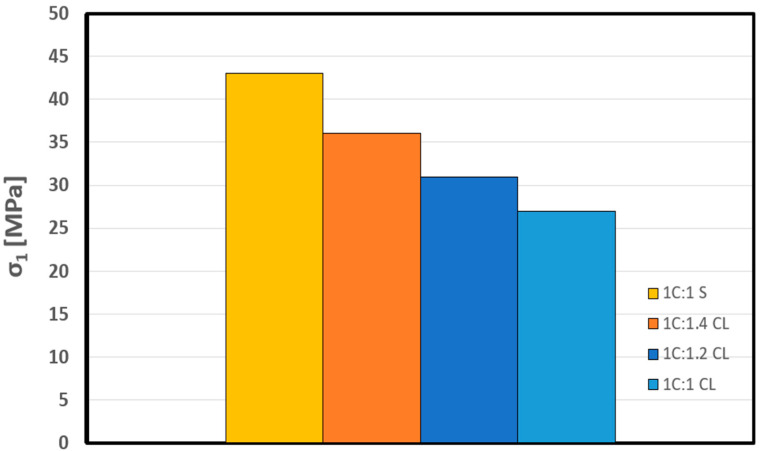
Variation of UCS in shotcrete samples.

In Table 4, the data clearly indicates that the uniaxial compressive strength (UCS) of the control mixture stands at a robust value of 43 MPa. Notably, when substituting the traditional sand component with crushed limestone, the strength of the shotcrete mixture exhibits a noticeable decline, a trend vividly illustrated in Figure 16. Nevertheless, it is crucial to emphasize that the UCS value demonstrates an intriguing sensitivity to variations in the crushed limestone (CL) ratio. This is elucidated by the fact that, with a 1C:1CL ratio, the UCS value decreases to 27 MPa. However, upon increasing the CL ratio to 1C:1.4 CL, there is a notable recovery in UCS, culminating in an improved value of 36 MPa. These findings underscore the dynamic relationship between the CL ratio and the uniaxial compressive strength, offering valuable insights into the optimization of shotcrete mixture performance.

Our analysis commences with a comprehensive investigation of stress–strain-based methods, where the standard deviation of these methods is observed to exhibit a range between 3.5 and 5.5, with a coefficient of variation spanning from 20% to 34.5%. These preliminary data establish the variability inherent in such methods, setting the stage for our subsequent investigations.

The focal point of our research centers on the average crack initiation (CI) value within the context of a control sample, designated as 1C:1S, where the four stress–strain-based methods are applied. As delineated in Table 4, the CI value for this control sample is quantified at 17 MPa. This benchmark value serves as a critical reference point for evaluating the impact of replacing sand with crushed limestone in the shotcrete mixture.

**Figure 17:**
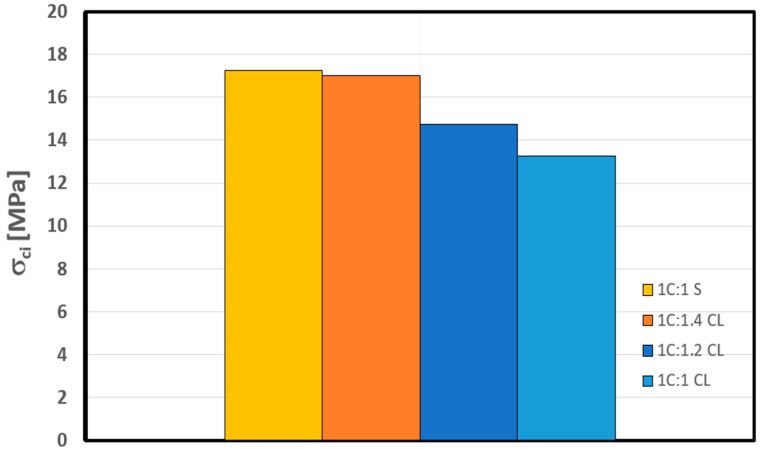
Variation of crack initiation in shotcrete samples.

Upon the replacement of sand with crushed limestone at an equivalent ratio, a notable decrease of 23% in the measured CI value is observed, reducing it to 13 MPa. This reduction in the CI threshold reduces the material resistance to crack initiation and, thus, increases its proneness to spall. Expanding our investigation further, an increase of 20% in the crushed limestone (CL) ratio to 1.2 results in a slight increment in the CI value, reaching 15 MPa (refer to Figure 17). Remarkably, when the CL ratio is raised to 1.4, the CI value also exhibits a subsequent increase to 17 MPa. This observation underscores an intriguing phenomenon: replacing sand in the shotcrete with a 1.4-to-1 ratio of crushed limestone to cement by dry weight appears to maintain a similar crack initiation threshold as the control sample. In other words, it maintains its resistance to in situ spalling failure.

Moreover, it is worth highlighting the correlation between the observed changes in CI and the unconfined compressive strength of the 1.4 CL ratio. Despite the reduction of the unconfined compressive strength to 36 MPa in comparison to the control sample, the crack initiation threshold remains analogous. This finding emphasizes the potential viability of crushed limestone as a replacement for sand in cement grout applications (i.e., shotcrete), especially when the in situ spalling failure risk is considered. This observation carries profound implications, particularly in the context of tunnel construction and maintenance, where the crack initiation threshold serves as a predictive indicator for spalling failure on tunnel sidewalls. Our study offers crucial insights into the feasibility of employing crushed limestone as an effective sand replacement, ultimately enhancing the structural performance and longevity of shotcrete in tunneling and underground infrastructure. The findings presented in this research promote safer and more durable construction practices while maintaining sustainability.

**Figure 18:**
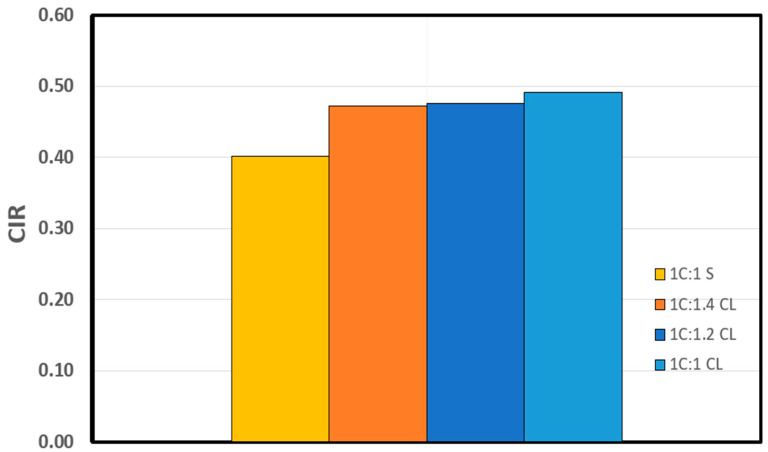
Variation of crack initiation ratio in cement grout samples.

The crack initiation ratio (CIR) serves as a dimensionless parameter, offering valuable insights into the prediction of in situ spalling failure. Typically, CIR values are within the range of 0.4 to 0.5 times the unconfined compressive strength (UCS) [16]. Previous studies show that the crack initiation ratio is independent of the material elasticity (i.e., elastic modulus and Poisson’s ratio) (see Table 2). The CIR, defined as the ratio of the uniaxial compressive strength ($\sigma_{1}$) to the average CI determined using four stress–strain-based methods for various grout mixtures, are presented in Table 4 and plotted in Figure 18. The baseline CIR for the control mixture stands at 0.40.

Upon replacing sand with crushed limestone within the shotcrete mixture, a discernible trend emerges, as vividly illustrated in Figure 18. A notable increase in the CIR is observed, particularly when adopting a 1C:1CL (cement to crushed limestone) ratio, where the CIR value increases to 0.49. Further investigation of the effect of varying the ratio of crushed limestone, reaching 1.4 per unit dry weight of cement, yielded a negligible decrease in CIR, with a value of 0.47. These findings lead to two significant observations. Firstly, the replacement of sand with crushed limestone in shotcrete is associated with a notable augmentation in the crack initiation ratio. Secondly, and rather intriguingly, the replacement ratio of crushed limestone within the shotcrete mixture seems to exert minimal influence on the crack initiation ratio. These observations underscore the sensitivity of the crack initiation ratio to the specific grout mixture material employed, while simultaneously highlighting the independence of the crack initiation ratio from alterations in the grout mixture ratios.

In summary, the empirical evidence presented in this study underscores the material-specific and ratio-independent nature of this crucial dimensionless parameter within the realm of shotcrete mixtures.

## 5. Summary and Conclusions

The demand for ores and advancements in drilling techniques have driven mining operations to unprecedented depths. These depths, combined with the challenging surrounding environment, which is characterized by high pressure and temperature and low porosity, bring forth significant risks—notably, rockburst and spalling failure. Spalling failure, in particular, involves the initiation of cracks within the elasticity zone of underground rock. While numerous crack initiation models exist, they are primarily developed based on the stress–strain response of underground rock. To mitigate these risks, shotcrete (i.e., cement grout)—a blend of cement, sand, water, and additives (when necessary)—is commonly used for tunnel support. However, the extensive use of natural sand in shotcrete mixtures raises environmental concerns. Sand extraction and transportation contribute to a significant carbon footprint, prompting a global shift towards more sustainable alternatives. Crushed limestone, a by-product of tunnel-boring activities, emerges as a promising candidate to replace natural sand in cement grouting for its sustainable attributes. Yet, the impact of crushed limestone on the critical crack initiation threshold, a pivotal factor in assessing shotcrete performance, remains underexplored.

With this context in mind, this study endeavors to investigate the potential of crushed limestone as a sustainable substitute for natural sand in shotcrete applications, particularly in deep tunnels. It aims to shed light on the implications of this substitution on the critical crack initiation threshold. To achieve this, uniaxial compressive tests on various shotcrete mixtures with three different cement-to-crushed limestone ratios (1:1, 1:1.2, and 1:1.4 by dry weight) were conducted. The crack initiation thresholds were determined using four stress–strain-based methods: the volumetric strain method, lateral strain method, extensional strain method, and Poisson’s ratio method.

The results showed a marked reduction in unconfined compressive strength as sand was replaced with crushed limestone in the shotcrete mixtures. However, an intriguing observation was that the unconfined compressive strength increased with higher crushed limestone content. Importantly, the study revealed that the crack initiation threshold exhibited a similar trend of change as sand was substituted with crushed limestone. Furthermore, an intriguing correlation was observed between the changes in crack initiation and the unconfined compressive strength of the 1.4 crushed limestone ratio. Despite the significant reduction in unconfined compressive strength compared to the control sample, the crack initiation threshold remained similar. This finding underscores the potential viability of using crushed limestone as a replacement for sand in cement grout applications, especially in mitigating in situ spalling failure risks in tunnel construction.

This observation holds profound implications, particularly in the realm of tunnel construction and maintenance, where the crack initiation threshold serves as a predictive indicator for spalling failure on tunnel sidewalls. The findings of this study offer critical insights into the feasibility of employing crushed limestone as an effective sand replacement, ultimately enhancing the structural performance and longevity of shotcrete in tunneling and underground infrastructure.

Moreover, the study delved into the crack initiation ratio (CIR), a dimensionless predictor of in situ spalling failure. It was observed that replacing sand with crushed limestone in the shotcrete led to a notable increase in the CIR. Surprisingly, the ratio of crushed limestone within the shotcrete mixture seemed to exert minimal influence on the CIR. These findings highlight the sensitivity of the CIR to the specific shotcrete mixture material employed, while also emphasizing the independence of the CIR from alterations in the shotcrete mixture ratios.

In summary, this research suggests that replacing sand with crushed limestone in cement grout mixtures reduces unconfined compressive strength and crack initiation stress while increasing the crack initiation ratio. By increasing the crushed limestone content in the cement grout from 1:1 to 1.4:1 of cement dry weight, both the unconfined compressive strength and crack initiation threshold increase, while the CIR remains relatively consistent. Of particular note is the similarity between the crack initiation stress of the 1:1.4 cement-to-crushed limestone mixture and the crack initiation of the control mixture. These findings provide valuable insights into determining the optimum proportion of crushed limestone required to replace sand in shotcrete mixtures, especially for supporting tunnel sidewalls that are susceptible to spalling failure. However, further studies are needed to explore durability, crack propagation mechanisms, optimal proportions, and practical implementation for tunnel sidewall support.

## Figures and Tables

**Table 3:** Designated shotcrete mixture.

Shotcrete Mix No.	Materials Ratio			
	Cement (C)	Crushed Limestone (CL)	Sand (S)	Water–Cement Ratio (*w*/*c*)
1	1	-	1	0.50
2	1	1	-	0.60
3	1	1.2	-	0.60
4	1	1.4	-	0.60

**Table 4:** Crack initiation stress and crack initiation ratio of the shotcrete samples.

$\sigma_{1}$ MPa	Shotcrete Mix	Crack Initiation Stress ($\sigma_{ci}$) [MPa]				SD	CoV (%)	Average of All CI Methods [MPa]	CIR
		Volumetric Strain Method	Lateral Strain Method	Extensional Strain Method	Poisson’s Ratio Method				
43	1C:1S	12	16	16	25	5.5	31.9	17.25	0.40
27	1C:1CL	10	12	11	20	4.6	34.5	13.25	0.49
31	1C:1.2CL	11	13	14	21	4.4	29.5	14.75	0.48
36	1C:1.4CL	16	14	16	22	3.5	20.4	17.00	0.47

## Data Availability

All data related to this manuscript are available upon request.
